# Targeting specific HATs for neurodegenerative disease treatment: translating basic biology to therapeutic possibilities

**DOI:** 10.3389/fncel.2013.00030

**Published:** 2013-03-28

**Authors:** Sheila K. Pirooznia, Felice Elefant

**Affiliations:** Department of Biology, Drexel UniversityPhiladelphia, PA, USA

**Keywords:** epigenetics, histone acetyltransferases (HATs), histone deacetylases (HDACs), neurodegenerative diseases, histone code, learning and memory, cell death, synaptic plasticity

## Abstract

Dynamic epigenetic regulation of neurons is emerging as a fundamental mechanism by which neurons adapt their transcriptional responses to specific developmental and environmental cues. While defects within the neural epigenome have traditionally been studied in the context of early developmental and heritable cognitive disorders, recent studies point to aberrant histone acetylation status as a key mechanism underlying acquired inappropriate alterations of genome structure and function in post-mitotic neurons during the aging process. Indeed, it is becoming increasingly evident that chromatin acetylation status can be impaired during the lifetime of neurons through mechanisms related to loss of function of histone acetyltransferase (HAT) activity. Several HATs have been shown to participate in vital neuronal functions such as regulation of neuronal plasticity and memory formation. As such, dysregulation of such HATs has been implicated in the pathogenesis associated with age-associated neurodegenerative diseases and cognitive decline. In order to counteract the loss of HAT function in neurodegenerative diseases, the current therapeutic strategies involve the use of small molecules called histone deacetylase (HDAC) inhibitors that antagonize HDAC activity and thus enhance acetylation levels. Although this strategy has displayed promising therapeutic effects, currently used HDAC inhibitors lack target specificity, raising concerns about their applicability. With rapidly evolving literature on HATs and their respective functions in mediating neuronal survival and higher order brain function such as learning and memory, modulating the function of specific HATs holds new promises as a therapeutic tool in neurodegenerative diseases. In this review, we focus on the recent progress in research regarding epigenetic histone acetylation mechanisms underlying neuronal activity and cognitive function. We discuss the current understanding of specific HDACs and HATs in neurodegenerative diseases and the future promising prospects of using specific HAT based therapeutic approaches.

## Introduction

The human genome encodes approximately 30,000 genes—but can this relatively fixed genome explain who we are or how we behave? A wealth of accumulating evidence suggests that there is much more to genome than its linear sequence of three billion basepairs. In fact, an additional level of “instructive” information superimposed on the DNA double helix in the form of a nucleoprotein entity termed the “chromatin” defines the three dimensional structure of the genome in the cell nucleus. The core unit of the chromatin is the nucleosome, which consists of 147 bp of DNA folded around histone octomers consisting two each of the histone proteins H2A, H2B, H3, and H4 (Berger, 2007). Although such organization of DNA in the form of chromatin allows packaging DNA within the constrained space of the nucleus, it also decreases the accessibility of DNA for key biological processes like transcription, replication, and repair. Despite the immense degree of global compaction, access to DNA is achieved by local chromatin decondensation in a highly regulated manner (Bonisch and Hake, 2012). While greater compaction of chromatin restricts accessibility, chromatin decondensation events generally allow for specific transcriptional regulator complexes to access DNA sequences, leading to enduring regulatory effects on gene expression and cellular function (Riccio, 2010). Such changes in chromatin compaction are mediated by stable and heritable modifications of both the DNA and its associated histone proteins that are independent of the underlying DNA sequence and together constitute the “epigenome” (“epi”—derived from Greek for “over” or “above”). Only a few years ago, the epigenome was primarily viewed in the context of cell division and early development wherein it serves to choreograph the myriad cellular and molecular events that promote specificity amongst various cell types that share a common genome within an individual. At first glance, these processes seemed to bear little relevance to the adult brain that is composed of a large proportion of post-mitotic and highly differentiated cells (Jakovcevski and Akbarian, 2012). However, recent explorations of the brain epigenome are providing unprecedented insights into the importance of specific epigenetic modification patterns in controlling gene expression not only in early brain development, but in adult brain functions as well, calling into place a “reprogramming process” that allows for plasticity at many levels of the neural circuitry in response to environmental cues (Borrelli et al., 2008). Together with reports implicating disordered chromatin organization and function in several age-related neurodegenerative diseases, these findings have in turn ignited enormous interest in examining how the course of normal maturation and aging affect the brain epigenome. While age related accumulation of somatic mutations and structural changes to the DNA are likely irreversible, most if not all of the epigenetic modification marks studied to date prove to be reversible. Thus, targeting the neural epigenome appears to be a promising strategy for neuroprotection and/or neuroregeneration both early in development as well as during the aging process (Jakovcevski and Akbarian, 2012). In this review, we will summarize recent progress in research linking epigenetic mechanisms, specifically histone acetylation to pathogenesis associated with age related neurodegenerative disorders. We will also discuss how this knowledge has the potential to be translated into suitable therapeutic strategies to treat these devastating conditions.

## Epigenetic modifications of DNA and histones

Epigenetics is historically defined as “the study of mitotically and/or meiotically heritable changes in gene function that cannot be explained by changes in DNA sequence” (Russo et al., 1996). This definition, however, is not particularly well suited for the nervous system where there is overall absence of mitosis. (Graff et al., 2011) therefore proposed a more recent definition for epigenetics as “the structural adaptation of chromosomal regions that allows to register, signal, or perpetuate altered activity states.” Such effects are primarily mediated *via* three major levels of epigenetic changes: (1) chemical modifications at the level of nucleotides that include DNA methylation and RNA interference (RNAi); (2) covalent post-translational modifications (PTMs) of histone proteins and incorporation of histone variants; and (3) nucleosome remodeling, referring to ATP-dependent processes that regulate the accessibility of nucleosomal DNA (Borrelli et al., 2008). Covalent histone modifications, histone variants, or chromatin remodeling complexes work together to alter the chromatin fiber, causing changes in the degree of chromatin compaction that correlate with “euchromatin” (open) vs. “heterochromatin” (closed) states (Cheung et al., 2000a; Strahl and Allis, 2000). These states generally align with active versus inactive states of gene expression, respectively (Berger, 2007). Numerous reviews on DNA methylation (Freitag and Selker, 2005; Miranda and Jones, 2007; Cedar and Bergman, 2012), non-coding RNAs (Bernstein and Allis, 2005), and ATP-dependent chromatin remodeling complexes (Ko et al., 2008; Hargreaves and Crabtree, 2011) have appeared in the literature. In this way, PTMs to histones and DNA act to regulate chromatin compaction that is critical in the control of both stable and transient gene expression profiles that dictate cell type specificity. Such epigenetic gene control mechanisms have more traditionally been viewed in the context of cell division and differentiation during early development. However, more recently, these same epigenetic mechanisms underlying gene control have been shown to work in the context of maintaining appropriate activity and function of post-mitotic neuronal cells, specifically in response to environmental stimuli. The current review will focus on covalent PTMs and in particular on recent findings implicating histone acetylation changes in the etiology of neurodegenerative diseases.

Histones are covalently modified at their amino terminal tails that extend beyond the globular core and undergo numerous PTMs which include in addition to the well studied acetylation and methylation, phosphorylation, ADP-ribosylation, sumoylation, ubiquitination, and proline isomerization (Peterson and Laniel, 2004). Remarkable progress has been made in characterizing the regulatory molecules that elicit such PTMs on the histone tails. Conceptually, these include the (1) Writers, enzymes that modify specific substrates by adding functional moieties like phosphate, acetyl or methyl groups; (2) Readers, regulatory proteins that share unique domains implicated in recognizing acetyl or methyl groups; (3) Erasers, enzymes that directly remove PTMs (Borrelli et al., 2008). Most PTMs target specific amino acid residues in the histone tails. For instance, phosphorylation is directed to serine and theronine residues, and methylation to arginines. However, lysines are targets for most modifications including acetylation and methylation. Moreover, covalently modified histones alone or in combination generate distinct docking sites and orchestrate the recruitment of multisubunit protein complexes that mediate cell- and promoter-specific gene expression. Histones are often concurrently modified on several residues and there is also a dynamic interplay between histone modifications and DNA modifications (such as DNA methylation), thus creating staggering combinatorial possibilities for gene regulation (Wood et al., 2006). For example, while phosphorylation of H3 Ser10 correlates with transcription activation in yeast (Lo et al., 2001), it also facilitates acetylation of H3 Lys14 in mammalian cells following stimulation by epidermal growth factor (EGF) with phosphorylation preceding acetylation (Cheung et al., 2000b). Furthermore, acetylation of H3 Lys 14 was found to be preferentially associated with EGF activated *c-fos* promoter in a MAP kinase-dependent manner, suggesting that interplay between acetylated and phosphorylated histone modifications may be involved in the expression of mitogen activated immediate early genes. In addition to its crosstalk with H3 Lys14, phosphorylation of H3 Ser10 has also been reported to play an important role in regulating H3 Lys9 methylation. In a study by Rea et al. (2000), methylation of H3 Lys9 was found to be significantly inhibited when a H3 tail peptide phosphorylated at Ser10 was used as a substrate in *in vitro* histone methyltransferase assays. Furthermore, acetylation and methylation of H3 Lys9 was found to be mutually exclusive. This observation is not surprising as acetylation of H3 Lys9 correlates with transcriptional activation, whereas methylation of histone H3 Lys9 correlates with gene silencing. On the contrary, methylation of H3 Lys4 localizes to sites of active transcription of chicken β-globin locus (Litt et al., 2001), suggesting that this modification may be stimulatory for transcription. Methylation of H3 Lys4 has also been shown to abrogate binding of the NuRD (Nucleosome remodeling and deacetylase) repressor complex and displacement of deacetylase activity of the NuRD repressor complex has been further postulated to facilitate acetylation of H3 Lys9 (Zegerman et al., 2002). These findings suggest that the controlled addition and removal of specific PTMs result in unique combinations that correspond to distinct physiological states and genomic functions.

## Decoding the epigenetic language in post-mitotic neuron function

Recent high resolution genome-wide profiling studies reveal that “epigenomes” are highly organized and strikingly non-random with respect to histone and DNA modifications (Bernstein et al., 2007). For example, high levels of H3 and H4 acetylation and H3 Lys4 trimethylation are generally present in promoter regions of active genes (Ruthenburg et al., 2007). In contrast, elevated levels of H3 Lys27 methylation correlate with polycomb protein mediated gene repression (Trojer and Reinberg, 2006). Interestingly, such epigenetic patterns vary in different cell types or during different stages of development (Trojer and Reinberg, 2006; Lessard et al., 2007; Putignano et al., 2007). More recently, specific chromatin signatures were also found at gene promoters, enhancers (Wang et al., 2008), and even exons (Andersson et al., 2009; Kolasinska-Zwierz et al., 2009; Spies et al., 2009; Tilgner et al., 2009). Moreover, individual PTMs can favor or inhibit consequent modifications on nearby residues of the same tail and examples of PTMs that influence modifications on different tails have also been reported (Sun and Allis, 2002; Latham and Dent, 2007). As lysines can be modified in various manners, it is the competition between various PTMs for the same residue that may determine functional outcomes (Zocchi and Sassone-Corsi, 2010). However, since distinct histone PTMs correlate with specific transcriptional states, it is conceivable that distinct histone modifications patterns on one or more tails are likely read like a molecular bar code to recruit chromatin remodeling complexes that drive gene expression profiles required for particular cellular events, a paradigm referred to as the “histone-code hypothesis” (Strahl and Allis, 2000; Fischle et al., 2003; Linggi et al., 2005).

Accumulating evidence also indicates that there also exists a “histone code” that regulates gene expression profiles for higher order brain functions like memory formation that requires the coordinated action of numerous signaling pathways to ultimately affect long term changes in gene expression (Agranoff, 1967; Flood et al., 1973). In mammalian associative memory tasks, activation of the ERK/MAPK (extracellular signal-regulated kinase/mitogen-activated protein kinase) signaling cascade in the hippocampus plays a crucial role in memory consolidation (Atkins et al., 1998). This process is typically accomplished by activation of the NMDA (N-methyl-d-aspartic acid) subtype of glutamate receptors, leading to an increase in intracellular Ca^2+^ (Fanselow et al., 1994). Ca^2+^ activates Ca^2+^-sensitive protein kinase C (PKC) and adenylyl cyclase/protein kinase A (PKA), thereby triggering a series of events that eventually converge upon ERK (Adams and Sweatt, 2002). Once activated, ERK translocates into the nucleus to coordinate and elicit changes in gene expression (Davis et al., 2000) by regulating transcription factors like CREB (Cre- binding protein) and Elk-1 (Davis et al., 2000) which in turn, initiate transcription of memory associated genes that contain their respective response elements (Chwang et al., 2006). An emerging model for effecting a stable, coordinated pattern of gene transcription underlying memory formation involves epigenetic tagging through modifications of histones. Accordingly, activation of NMDA receptors and subsequently, the ERK/MAPK signaling cascade has also been reported to result in a transient increase in both H3 Ser10 phosphorylation and H3 Lys14 acetylation in the CA1 region of rat hippocampus following contextual fear conditioning, a test routinely used to assess associative learning and long term memory formation (Chwang et al., 2006). In this paradigm, peak level of these modifications was observed 1 h post conditioning, corresponding to the period when rapid hippocampal gene induction occurs (Levenson et al., 2004). Furthermore, such H3 modifications were abolished when memory formation was impaired by blockade of NMDA receptors or by a latent inhibition paradigm, suggesting that these modifications are specifically associated with memory consolidation. Thus, it seems like phosphorylation and acetylation of histone H3 may serve as part of a histone combinatorial code that is subsequently interpreted as a pattern of gene expression specific to contextual fear conditioning memory. Recent studies have also identified DNA methylation, once thought to be a static process after cellular differentiation, to work in concert with H3 acetylation to dynamically regulate plasticity and memory formation in adult rat hippocampus following contextual fear conditioning (Miller and Sweatt, 2007; Miller et al., 2008). But how do such combinatorial histone modifications affect memory formation? (Wood et al., 2006) proposed that histone modifications may gate a burst of transcription for a specific set of plasticity effector and regulator genes that then change the response properties of individual neurons in a network. Histone modifications may also mediate persistent changes in the expression of key plasticity effector or regulator genes required for maintenance of changes in neuronal behavior. It is likely that transient histone modifications may act downstream of signaling cascades to integrate multiple signals and ensure that a cascade of gene expression is activated only after a particular stimulus pattern (either spatially or temporally) is generated (Schreiber and Bernstein, 2002). Under such conditions, histone modifications may act to integrate information about the activation and regulate recruitment of process specific transcription factors. Thus, specific histone modification patterns not only serve to alter the chromatin structure but also provide an interaction interface for transcriptional co-activators or co-repressors that bind modified histone tails to regulate specific transcription events (Wood et al., 2006). However, studies aimed at deciphering the “epigenetic indexing code” specific for high-order brain functions like memory formation are still in their infancy. An increased understanding of chromatin function and epigenetic tagging may further help delineate the role of particular epigenetic mechanisms in brain functions in more molecular detail.

## Epigenetics based activity-dependent plasticity in brain function

Phenotype is the net result of continued gene—environment interactions. Environmentally regulated intracellular signals “program” regulated expression of very specific gene sets that are required for the development and function of specific cell lineages (Tilgner et al., 2009). In the nervous system, the mechanisms by which extracellular signals regulate gene expression have just begun to be characterized. Indeed, epigenetic modifications, such as DNA methylation and PTMs of histone proteins are emerging as fundamental mechanisms by which neurons adapt their transcriptional response to developmental and environmental cues. The implicit hypothesis is that environmental signals alter such chromatin modifications, allowing for the transcriptional “plasticity” that in turn mediates sustained variation in neural function (Meaney and Ferguson-Smith, 2010). In support of this concept, Nelson et al. (2008) reported that spontaneous synaptic transmission in hippocampal neurons is regulated by alterations in DNA methylation that occur in response to synaptic activity. Moreover, treatment of mouse E14 (embryonic day14) cortical cultures with KCl that induces membrane depolarization enhances transcription of brain derived neurotrophic factor (BDNF) that is critical in promoting adult neural plasticity associated with learning and memory (Martinowich et al., 2003). Such an effect was found to be associated with decreased CpG methylation within exon IV promoter of the *Bdnf* gene as well as dissociation of the MeCP2 (methyl cytosine binding protein 2)- HDAC 1 (histone deacetylate 1)-mSin3A repressor complex from its promoter. This suggests that DNA methylation–related chromatin remodeling may play a crucial role in activity-dependent gene regulation critical for neural plasticity (Martinowich et al., 2003).

In the hippocampus, various signaling pathways involving dopaminergic, acteylcholinergic, and glutamatergic signaling have been implicated in synaptic plasticity *via* activity-dependent epigenetic mechanisms of neuronal gene regulation [reviewed in Riccio (2010)]. Glutamate is a primary neural signal for synaptic plasticity, and both glutamate as well as direct activation of its NMDA receptor induces MeCP2 phosphorylation on Serine 421 in cultured hippocampal neurons (Zhou et al., 2006). Such activity-dependent site specific phosphorylation of MeCP2 has more recently been shown in a mouse model to be required for MeCP2 genome-wide recruitment that serves to facilitate experience-dependent chromatin remodeling (Cohen et al., 2011). Activity-dependent gene regulation is also mediated by site specific chromatin acetylation although the full array of HATs that create these marks and the mechanisms by which they respond to environmental stimuli is only recently emerging. One of the best characterized of these HATs is CBP, shown to utilize Ca^2+^-dependent signaling pathways to link its action to environmental cues. In an elegant study using primary mouse neuronal cultures exposed to elevated levels of potassium chloride (KCl), (Kim et al., 2010) demonstrated that Ca^2+^ influx specifically *via* L-type voltage-sensitive calcium channels triggers widespread transcription at activity-dependent neuronal enhancers *via* site-specific recruitment of CBP to these genomic locations. Neuronal activity also regulates chromatin acetylation by controlling the shuttling of certain class II HDACs in and out of the nucleus by both NMDA receptor mediated Ca^2+^ influx-dependent mechanisms and by direct electrical activity. Such control of HDAC intracellular localization plays a key role in modulating chromatin acetylation and transcriptional activity of neuronal genes in response to environmental stimuli [reviewed in Riccio (2010)].

Sensory experiences in the form of neuronal activity also have differential effects on synaptic plasticity at excitatory or inhibitory synapses, leading to either long term potentiation (LTP) or long term depression (LTD), whereby the efficacy of synaptic transmission is up- or down-regulated, respectively (Borrelli et al., 2008). Certain forms of LTP and LTD require long-lasting changes in gene expression and a growing body of evidence suggests that histone PTMs may be involved in these processes. In an elegant study using sensory motor neurons of *Aplysia*, Guan et al. (2002) showed that an increase and decrease of acetylated histones might constitute the switch between LTP and LTD at the same synapses. This study demonstrated that within a single sensory neuron, the excitatory neurotransmitter serotonin induces expression of CREB1 transcription factor which in turn recruits the HAT CBP (CREB binding protein). Subsequently, through histone acetylation and the recruitment of transcriptional machinery, CREB1/CBP together lead to activation of the downstream gene C/EBP (CCAAT enhancer binding protein) that is required for long-term synaptic plasticity with increased synapse strength (LTP). On the contrary, treatment of these sensory neurons with an inhibitory transmitter FMRFamide causes displacement of CREB1/CBP with the repressor complex CREB2(ATF4)/HDAC5 on the target C/EBP gene promoter, leading to promoter deacetylation and inhibition of C/EBP gene expression as well as subsequent switch of synaptic plasticity into LTD (Guan et al., 2002).

The proper execution of complex animal functions and their breakdown in disease involves an interaction between the genetics of an animal and its environment (Arai and Feig, 2011). In a study by Greenough et al. (1972), it was demonstrated that environmental enrichment (EE) in the form of availability of a wide variety of toys, exercise apparati, and socially complex housing, boosts memory capacity in mice. Novel stimuli such as that induced by EE have been observed in different animal models to induce a natural exploration behavior and increase the release of dopamine in hippocampus and prefrontal cortex (PFC) (Ljungberg et al., 1992; Ihalainen et al., 1999; Li et al., 2003). Dopaminergic innervation is critical for long term changes in synaptic efficacy in hippocampus and PFC (Gurden et al., 2000; Li et al., 2003; Huang et al., 2004; Granado et al., 2008), as well as for learning-associated immediate-early gene expression (Lisman and Grace, 2005; Granado et al., 2008). In a recent study by Sarantis et al. (2012), it was demonstrated that exposure of rats to a novel open-field environment that increases their exploratory behavior evokes dopamine release in the hippocampus and PFC. Furthermore, this spatial novelty leads to chromatin remodeling events characterized by histone H3 Ser10 phosphorylation and H3 Lys14 acetylation with concomitant increase of the IEGs c-Fos and zif/268 protein expression in the CA1 region of the hippocampus. Both these events are also dependent upon phosphorylation of NMDA and AMPA (α-amino-3-hydroxy-5-methyl-4-isoxazole propionic acid) receptor subunits and subsequent activation of the ERK signaling pathway which as described earlier mediates neuronal synaptic plasticity. By using CK-p25 Tg mice that allow temporally and spatially restricted induction of neurodegeneration, Fischer et al. (2007) showed that EE reinstates associative and spatial learning in these mice with severe neurodegeneration and promoted growth of new dendrites and synapses in the hippocampus. Furthermore, (Fischer et al., 2007) trained a group of these animals using fear conditioning, a learning method by which organisms learn to associate a neutral stimulus with another, unpleasant stimulus. They then allowed the animals' memory for that training event to decay over time (directly or indirectly through neurodegeneration), and confirmed that the animals had lost the capacity to recall that memory. Remarkably, the ability of the animals to recall that memory, which had apparently been lost, was restored by EE. Such an effect was also associated with increased hippocampal acetylation of histones H3 and H4. Accordingly, administration of the HDAC inhibitor Sodium butyrate restores associate and spatial learning in the CK-p25 Tg mice similar to EE. Together, these studies suggest that EE might mediate “rewiring” of the neural network that underlies memory formation through chromatin remodeling.

With identification of nuclear enzymes that regulate histone PTMs (like acetylation, lysine/argine methylation, phosphorylation, deamination, ubiquitination), it is conceivable that most, if not all, chromatin modifying enzymes are targeted by signaling pathways that directly link environmental cues to gene expression (Wood et al., 2006). Nevertheless, the complete repertoire of extracellular signals and corresponding intracellular pathways that mediate dynamic regulation of histone modifications in neurons remains poorly understood.

## Alterations of the brain epigenome as part of aging and in neurodegenerative diseases

**Figure 1:**
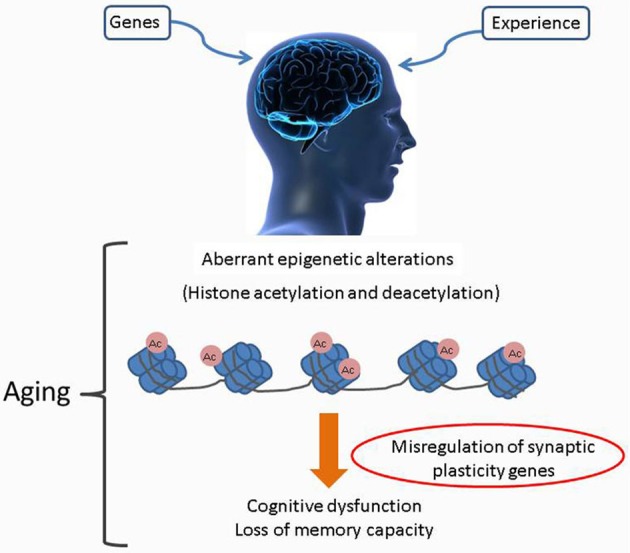
**Age associated alterations to the brain epigenome.** Age associated cognitive decline as caused by accumulated alterations of histone acetylation patterns within the brain epigenome. Misregulation of specific HAT production and/or their targeting to chromatin leads to complex changes in the chromatin landscape with subsequent altered transcription profiles. Such negative changes exacerbate an individual's vulnerability to age related cognitive decline.

An increasing body of evidence indicates that substantial reorganization of the brain epigenome occurs during aging and such “age related” epigenetic drift could further exacerbate an individual's vulnerability to “aging related” cognitive decline (Graff and Mansuy, 2009; Penner et al., 2010). This notion that aging is associated with epigenetic changes in the brain is substantiated with studies reporting widespread age-related changes in gene expression in the cerebral cortex, including downregulation of many neuronal genes (Erraji-Benchekroun et al., 2005; Tang et al., 2009), global loss of DNA methylation in aging, or the hypermethylation of regulatory regions (promoters) of genes associated with accelerated aging (Tang et al., 2009; Gonzalo, 2010; Han and Brunet, 2012; Winnefeld and Lyko, 2012). In addition, dynamic changes to the epigenetic landscapes of PTMs can also occur and are characterized by loss of markings associated with active gene expression, such as monomethylation of H4 Lys20 and trimethylation of H3 Lys36, in conjunction with robust increase in the repressive mark H3Lys27me3 (Wang et al., 2010). Likewise, in the hippocampi of 16-month old wild type mice, genomic regions associated with actively expressed genes shows a decline in acetylated H4Lys12, a PTM linked to transcription elongation (Peleg et al., 2010). It is likely that such age-related drifts in brain epigenomes negatively affect neuronal and oligodendroglial transcriptomes, thereby leading to a decline in signaling capacity of nerve cells (Lu et al., 2004; Copray et al., 2009; Fischer et al., 2010). With regards to specific neurophysiological processes, it is well-established that memory and synaptic plasticity processes in the cognitively healthy adult require transcription of immediate-early genes (IEGs), including *Arc* (activity-regulated cytoskeletal gene), *zif268* (also known as nerve growth factor inducible-A), and *bdnf* (brain-derived neurotrophic factor) (Guzowski et al., 2000; Hall et al., 2000; Steward and Worley, 2001). While blocking the expression of these genes in adult animals prevents the consolidation of memory (Linnarsson et al., 1997; Guzowski et al., 2000), decreased IEG expression is also prevalent in many models of memory disorders (Dickey et al., 2003; Palop et al., 2005; Rosi et al., 2005) and as a result of the normal aging process (Blalock et al., 2003; Small et al., 2004; Rowe et al., 2007). Accumulating evidence indicates that epigenetic mechanisms play a key role in dynamically regulating memory associated gene transcription in the adult CNS and are thus integral to long term memory formation (Levenson and Sweatt, 2005; Lubin et al., 2008). In light of studies reporting a decline in the transcription of key memory-promoting genes during aging (Blalock et al., 2003; Copray et al., 2009; Winnefeld and Lyko, 2012) it has been hypothesized that such changes could be mediated by dysregulation of epigenetic control mechanisms over the lifespan of an individual. Consequently, accumulation of aberrant epigenetic marks within brain regions vulnerable to the aging process may result in age-related cognitive deficits and are also manifested in the form of neurodegenerative diseases (Penner et al., 2010) (Figure 1).

Age-related neurodegenerative disorders such as Huntington's disease (HD), Alzheimer's disease (AD), Parkinson disease (PD), amyotrophic lateral sclerosis (ALS), and others are multifactorial illnesses in which many as yet poorly understood pathways are affected serially and in parallel, resulting in pathologic phenotypes like cognitive decline. Recent studies have linked phenotypic as well as mechanistic features common with this array of neurodegenerative diseases to epigenetic defects (Kwok, 2010; Marques et al., 2011; Babenko et al., 2012). In support of this concept, both familial and sporadic forms of AD, PD, and ALS are known to occur, familial forms represent only a minority of the cases and the vast majority of cases occur as sporadic forms that are likely to result from complex interactions between genetic and environmental factors that superimpose on the slow, sustained neuronal dysfunction due to aging, a major risk factor for neurodegenerative diseases (Migliore and Coppede, 2009). In fact, “synucleopathies” such as Parkinson's disease and dementia with Lewy bodies are associated with dysregulation of DNA methylation at the promoters of several disease-associated genes, an effect primarily mediated by cytoplasmic sequestration of DNA methyltransferase 1(Dnmt1) by α-synuclein that results in a decrease in nuclear Dnmt1 (Desplats et al., 2011). Histone modifying enzymes have also been implicated in neurodegenerative diseases. For example, the pathological sequestration of transcription factors vital for neuronal health, such as the cAMP response element-binding protein CREB and its binding partner CBP, a histone acetyltransferase (HAT), has been linked to the beta amyloid plaques seen in the brains of individuals with Alzheimer's disease (AD) (Tong et al., 2001; Vitolo et al., 2002; Caccamo et al., 2010). Sequestration of CBP within nuclear polyglutamine inclusions that results in a decrease in soluble CBP and CBP-dependent transcription has also been observed in cell culture and transgenic mouse models of polyglutamine disorders like spinocerebellar ataxia type 3 (McCampbell et al., 2000; Chai et al., 2002), and Huntington's disease (Steffan et al., 2001). The HAT, Tip60 has been reported to interact with ataxin 1 protein in Spinocerebellar ataxia 1 (SCA1) mouse model and contribute to cerebellar degeneration associated with SCA1, a neurodegenerative disease caused by polyglutamine tract expansion in the ataxin-1 protein (Gehrking et al., 2011). Furthermore, excessive H3Lys9 methylation (Ryu et al., 2006) and increased expression of macro H2A1, a variant histone broadly associated with repressive chromatin (Hu et al., 2011), have been observed in blood and brain tissues from individuals with Huntington's disease in brains regions like the striatum and cerebral cortex which are heavily affected by the disease associated neurodegenerative process (Jakovcevski and Akbarian, 2012). These studies highlight the fact that epigenetic mechanisms may be crucial to advancing our understanding of how individual differences modulate susceptibility to neurodegenerative diseases. Originally thought to be stable and irreversible, epigenetic mechanisms have been demonstrated by several recent studies to be dynamic and reversible even in fully differentiated post-mitotic brain cells. This reversibility supports the development of epigenetic-based pharmacological interventions to alleviate or reverse the symptoms resulting from their dysfunctions (Graff and Mansuy, 2009).

## Histone acetylation: a key epigenetic modification for neuronal survival and function

### HAT: HDAC imbalance in the etiology of neurodegenerative diseases

In neurons, histone acetyltransferases (HATs) and histone deacetylases (HDACs) are among the best characterized chromatin modifying enzymes and represent distinct classes that, respectively, catalyze forward and reverse kinetics of lysine residue acetylation in specific histone substrates. HATs function enzymatically by transferring an acetyl group from acetyl-coenzyme A to the ∈-amino groups of histone lysine residues thereby creating a specific “histone code” for chromatin modification that in general, enhances DNA accessibility for transcription factor binding. Contrarily, HDACs attenuate transcription levels by deacetylating such lysine targets (Legube and Trouche, 2003). Under normal conditions, maintaining the balance between HAT and HDAC levels and activity is crucial for establishing appropriate histone modification patterns that serve to regulate both stable and rapidly changing gene expression profiles critical for both neuronal homeostasis, and appropriate neurophysiological response outputs such as long-term potentiation, learning, and memory, respectively (Saha and Pahan, 2006). In support of this concept, treatment of the dopaminergic neuronal cell lines like rat N27, mouse MN9D, and human SH-SY5Y cells with the HDAC inhibitor trichostatin A (TSA) under normal conditions has been found to induce neuronal apoptosis (Wang et al., 2009). Similarly, overexpression of CBP in resting primary cerebellar granule neurons (CGN) under prosurvival conditions leads to chromatin condensation and cell death (Rouaux et al., 2003). Neuronal overexpression of Tip60 also leads to increased apoptosis and lethality in *Drosophila* (Pirooznia et al., 2012b). Such lethal effects are likely mediated by skewing the HAT/HDAC balance toward increased acetylation that in turn brings about alterations in the chromatin structure that leads to activation/de-repression of genes that are quiescent under basal conditions. On the contrary, induction of apoptosis in CGN primary cultures by neurotrophic deprivation leads to H3 and H4 deacetylation that precedes neuronal death and is also accompanied by loss of CBP, an effect mediated by degradation of CBP by caspase-6 (Rouaux et al., 2003). Together, these studies support the maintenance of optimal HAT/HDAC balance for neuronal survival, notably in differentiated adult neurons that have to maintain their functional status and homeostasis throughout their lifetime.

Consistent with the above studies, altered levels of histone acetylation have also been observed in several models of neurodegenerative diseases. For instance, toxic accumulation of α-synuclein in the nucleus of dopaminergic neurons induces neurotoxicity by promoting H3 deacetylation through direct association with histones thereby shielding residues targeted for acetylation (Kontopoulos et al., 2006). The polyglutamine disease protein ataxin-3 has also been reported to cause transcriptional repression by binding histones H3 and H4 thereby blocking access to acetylation sites on these histones. Additionally, ataxin-3 also binds coactivators like CBP, p300, and CBP/p300 associated factor (PCAF) and represses the respective coactivator mediated transcription (Li et al., 2002). Expression of the polyglutamine-containing domain of the pathogenic Huntington (Htt) protein in cultured cells (PC12) also leads to H3 and H4 deacetylation (Steffan et al., 2001; Ferrante et al., 2003; Jiang et al., 2006). In an ALS mouse model (SOD1 G86R), H3 hypoacetylation has been observed in cholinergic motor neurons from the lumbar spinal cord (Rouaux et al., 2003, 2007). While these studies identify histone deacetylation as a common feature of neurotoxicity under pathological conditions, as mentioned above, histone hyperacetylation can also be fatal to neurons. In a study by Song et al. (2010), it was reported that Dieldrin, a neurotoxic peptide implicated in the etiopathogenesis of PD, induces a time-dependent accumulation of CBP, resulting in increased H3 and H4 acetylation in dopaminergic neurons. Together, this series of studies strongly point toward a loss of neuronal acetylation homeostasis during neurodegeneration. How can impairment of acetylation homeostasis lead to neuronal loss? The clue to this question revolves around the hypothesis of “transcriptional dysfunction” that attributes the degenerative fate of neurons to altered transcription profiles resulting from complex changes in the chromatin histone acetylation landscape that differs sharply from activity-dependent normal modification and transcription patterns. As a result, expression of pro-survival associated genes is likely attenuated by such alterations, while expression of pro-apoptotic genes is induced. Such gene expression alterations consequently lead to neuronal cell death, a major pathological hallmark of many neurodegenerative diseases (Saha and Pahan, 2006). However, neuronal cell death and activation of apoptotic pathways associated with loss of neurons is a late event in the disease associated pathogenesis (Brady and Morfini, 2010).

Accumulating evidence indicates that the clinical symptoms associated with neurodegenerative diseases are the result of the accumulation of early and subtle neuronal dysfunction that precedes actual cell demise and is manifested through loss of synaptic connectivity. For instance, in Alzheimer's disease (AD), synaptic degeneration appears to be an early event in pathogenesis with synapse loss evident in patients with early AD and mild cognitive impairment (Scheff et al., 2007; Arendt, 2009). Accordingly, it has been proposed that synapse loss underlies memory impairment evident in the early phase of AD (Shankar and Walsh, 2009). Recent studies propose that changes in histone acetylation levels may be involved in the altered synaptic function and memory associated with AD (Sananbenesi and Fischer, 2009; Xu et al., 2011). Consistent with this hypothesis, pre-clinical studies in APP/PS1 mouse model of AD have reported differences in histone acetylation levels during associative memory formation wherein levels of hippocampal acetylated histone H4 in APP/PS1 mice were about 50% lower than in wild-type littermates after fear conditioning training (Francis et al., 2009). Likewise, in HD, there is now considerable evidence that early cognitive deficits appear in patients before the onset of the characteristic motor disturbances (Van Raamsdonk et al., 2005). Early impairment of long-term spatial and recognition memory in heterozygous HD knock-in mutant mice (HdhQ7/Q111) is also associated with reduced hippocampal activity of CBP and diminished levels of histone H3 acetylation with concomitant reduction in expression of memory related genes (Giralt et al., 2012). These studies further support the notion that disruption of acetylation homeostasis can lead to early and widespread synaptic dysfunction likely resulting from impairment of transcriptional profiles critical for promoting appropriate neuronal connectivity. Accumulation of these early and often subtle defects likely ultimately lead to neuronal apoptotic cell death.

### HAT: HDAC interplay in memory formation

A number of recent studies have identified histone acetylation as an essential mechanism for formation of long-term memories (Levenson and Sweatt, 2005). For instance, associative learning in rats induces a transient increase in hippocampal acetylation of histone H3 but not H4 1 h after training (Levenson et al., 2004). A recent study also reported an increase in acetylation of histones H3 Lys9, Lys14 and H4 Lys5, Lys8, Lys12 (but not Lys16) 1 h after contextual fear conditioning in healthy young mice, Peleg et al. (2010) suggesting that this type of memory formation leads to very specific re-organization of the chromatin structure. Similar changes in histone acetylation modification patterns have been observed in other hippocampus-dependent learning paradigms and are restricted to periods where critical phases of transcription associated with long-term memory formation occurs [reviewed in Graff et al. (2011)]. The above mentioned study by Peleg et al. (2010) also reported that while aged mice that exhibited memory disturbances displayed a transient increase in H3 Lys9, Lys14 and H4 Lys5, Lys8 acetylation 60 min after fear conditioning, there was no change in acetylation of histone H4 at lysine12 (H4K12) in response to learning. Furthermore, the specific lack of H4K12 acetylation correlated with a severely impaired hippocampal gene expression program required for memory formation. By analyzing the distribution of H4K12 acetylation in young and aged mice during learning, it was determined that impaired H4K12 was selectively associated with the coding regions of genes that are normally upregulated during learning. Accordingly, restoration of physiological H4K12 acetylation reinstated the expression of learning-induced genes and led to the recovery of cognitive abilities. Together, these studies provide convincing evidence in favor of a casual role for histone acetylation in mediating gene expression changes associated with consolidation of long term memory as well as age-associated memory impairment.

Recent studies have also identified specific HATs and HDACs that are required for memory formation, and deregulation of such enzymes have also been linked to age-associated memory impairment (Fischer et al., 2010). To this end, several genetic studies have identified the HAT CBP as a major contributor to memory formation (Barrett et al., 2011). Mice haploinsufficient for CBP (cbp±) exhibit reduced acetylation, defects in hippocampal late long-term potentiation (L-LTP), and some forms of long-term memory (LTM) deficits (Alarcon et al., 2004). Importantly, the HAT activity of CBP was shown to be required for these processes (Korzus et al., 2004). In addition, other HATs like the E1A-binding protein p300 (p300) and p300/CBP-associated factor (PCAF), have also been implicated in memory processes (Oliveira et al., 2007; Maurice et al., 2008). PCAF homozygous knock-out (KO) mice are viable and display short term memory impairments at adolescent age (2 months). However, memory impairments observed in the PCAF KO mice change with age toward contextual long-term memory deficits at 6 and 12 months (Maurice et al., 2008). In addition, learning induced upregulation of CBP, p300, and PCAF has also been associated with elevated H2B and H4 acetylation during spatial long-term memory consolidation (Bousiges et al., 2010). This is consistent with previous studies that showed that learning increases hippocampal H2B and H4 acetylation (Koshibu et al., 2009; Peleg et al., 2010). Together, these studies suggest that HATs exhibit certain substrate specificity during memory formation in the adult brain and mediate dynamic acetylation of such substrates. Interestingly, histone acetylation at certain residues may influence the recognition and binding of certain HATs that may then promote acetylation of additional residues. In support of this concept, the crystal structure of the Yeast Gcn5 bromodomain revealed that it may discriminate between binding to different acetylated lysine residues on histone H4 depending upon the context in which they reside (Owen et al., 2000). Interestingly, H4K16 is the only histone modification that is not regulated during memory consolidation in mice, while exposure of mice to associative learning increases hippocampal H4K5, H4K8, and H4K12 acetylation as well as H3K9 and H3K14 acetylation (Peleg et al., 2010). Thus, H4K16 is likely at the base of the pyramid of H4 acetylation and in turn mediates acetylation of nearby lysine substrates in a process specific manner (Stilling and Fischer, 2011). A recent study reported that HATs like CBP, p300 and PCAF that all harbor a bromodomain are upregulated during spatial memory formation while the Tip60 that lacks a bromodomain was not upregulated (Bousiges et al., 2010). Similarly, in a recent gene array study, the bromodomain containing HATs, Taf1/Kat4, Gcn5/Kat2a were found to be upregulated 1 h after a fear conditioning stimulus (Peleg et al., 2010). Together, these findings suggest a model wherein a stimulus driven upregulation of bromodomain containing HATs induce histone acetylation that is required for transcription of plasticity-related genes (Stilling and Fischer, 2011).

Histone acetylation is mediated by the concerted actions of HATs and HDACs (Legube and Trouche, 2003). The mammalian genome encodes 11 HDAC proteins consisting of the class I (HDACs 1, 2, 3, and 8), class II (HDACs 4, 5, 6, 7, 9, and 10), class III sirtuins (SIRT 1, 2, 3, 4, 5, 6, and 7), and class IV (HDAC 11) HDACs (Thiagalingam et al., 2003). With regards to memory formation, HDAC2 was recently shown to be associated with promoters of genes implicated in synaptic plasticity including *Egr1* (also known as *zif*268), *Bdnf*, *Fos*, and *Creb*. Accordingly, neuronal overexpression of HDAC2 in mice, but not that of HDAC1, decreased dendritic spine density of hippocampal CA1 pyramidal neurons and dentate gyrus granule cells, impaired hippocampus-dependent synaptic plasticity and suppressed the expression of synaptic remodeling and plasticity genes, indicating that HDAC2 negatively regulates memory formation (Guan et al., 2009). Conversely, HDAC2 knock-out mice exhibit enhanced memory formation that correlated with elevated H4K12 acetylation which as mentioned above has been implicated in gene expression programs required for memory formation. Similar to HDAC2, specific deletion of HDAC3 in the dorsal hippocampus of mice leads to enhanced long term memory and elevated expression of Nr4a2, a gene associated with long term memory formation (McQuown et al., 2011). This series of studies identifying the role of specific HATs and HDACs in memory formation highlight the crucial dependency of long term memory formation on these key epigenetic players.

## Targeting histone deacetylases: epigenetic strategy for neurodegenerative diseases

The above studies identifying a critical role for histone acetylation in promoting neuronal cell survival and memory formation support recent findings demonstrating that deregulation of histone acetylation is causatively linked to the pathogenesis of various neurodegenerative diseases (Stilling and Fischer, 2011). In light of these studies, the use of histone deacetylase inhibitors (HDACi) as a therapeutic tool for neurodegenerative disorders has been examined with great interest in the last decade (Dietz and Casaccia, 2010). Histone acetylation changes in specific brain regions like the hippocampus, amygdala, and medial prefrontal cortex have also been implicated in the persisting abnormalities of stress-related psychopathology like depression (Tsankova et al., 2007; Covington et al., 2009), anxiety disorders (McEwen et al., 2012), and schizophrenia (Tang et al., 2011). Accordingly, local administration of HDACi in such brain regions has been observed to have antidepressant-like actions in several behavioral assays (Schroeder et al., 2007; Bredy and Barad, 2008; Grayson et al., 2010). These studies suggest that HDACi show potential as anti-depressant agents. The potential contribution of epigenetic mechanisms including histone acetylation to susceptibility to stress related disorders and the reversal of disease symptoms by HDACi has been extensively reviewed in the literature (Abel and Zukin, 2008; Sun et al., 2013; Vialou et al., 2013). This section will therefore review some of the recent data linking dysregulation of specific HATs and HDACs to neurodegenerative diseases as well as the promising effects observed with HDACi in preventing cell death and alleviating disease associated pathological symptoms.

### Huntington's disease

Huntington's disease (HD) is an inherited genetic disorder, caused by an abnormally expanded and unstable CAG repeat (polyglutamine or polyQ expansion) within the coding region of the gene encoding the huntington (Htt) protein. One of the models for mutant huntington protein induced toxicity is based on the finding that a polyglutamine peptide encoded by the first exon of Htt (Httex1p) directly binds the acetyltransferase domains of CBP and PCAF *in vitro* (Steffan et al., 2001; Cong et al., 2005). This appears to sequester these acetyltransferases, resulting in globally reduced H3 and H4 acetylation levels, and altered gene expression (Steffan et al., 2001). Overexpression of the expanded HD constructs has been shown in different cellular models to cause redistribution of CBP in nuclear or cytoplasmic inclusions. This phenomenon is accompanied by inhibition of HAT activity of CBP, further leading to global deacetylation and cell death (McCampbell and Fischbeck, 2001). Mutated polyQ-expanded Htt has also been shown to selectively enhance ubiquitylation and degradation of CBP (Jiang et al., 2003; Cong et al., 2005). Treatment with HDAC inhibitors (HDACi) have been reported to rescue histone acetylation levels and improve neurodegeneration and pathological symptoms in cellular, *Drosophila* and mouse models of HD. For instance, expression of HTTex1p in cultured PC12 cells reduces H3 and H4 acetylation levels, an effect that can be reversed by administration of HDACi like trichostatin (TSA) and suberoylanilide hydroxamic acid (SAHA) (Steffan et al., 2001). Neuronal expression of the expanded repeat Httex1p in *Drosophila* has been observed to be intrinsically cytotoxic, resulting in reduced viability and degeneration of photoreceptor neurons. However, treating these flies with the HDACi Sodium butyrate and SAHA was found to increase viability and prevent progressive degeneration of photoreceptor neurons (Steffan et al., 2001). Httex1p induced neurodegeneration of photoreceptor neurons in *Drosophila* can also be suppressed by genetic or pharmacological reduction of the class I HDAC, Rpd3, and the class III HDAC, sirtuin 2 (Sir2) either individually or in combination (Pallos et al., 2008). Administration of the pan-HDACi, suberoylanilide hydroxamic acid (SAHA) has been shown to increase histone acetylation and improve motor impairment in the R6/2 transgenic HD mouse model (Hockly et al., 2003). Such an effect has been reported to be mediated via reduction of HDAC2 and HDAC4 by SAHA in the cortex and brain stem of R6/2 mice (Mielcarek et al., 2011). In the same R6/2 mice model, presymptomatic intraperitoneal administration of another pan-HDACi, sodium butyrate extended survival and prevented striatal neuronal atrophy with resultant improvement in motor performance (Ferrante et al., 2003). A novel pimelic diphenylamide HDACi, 4b, has also shown beneficial effects on disease phenotype and transcriptional abnormalities in an HD mouse model (Thomas et al., 2008). Together, these studies support the notion that HD is a disease of aberrantly reduced histone acetylation.

### Parkinson's disease

Parkinson's disease (PD) is a progressive neurodegenerative disorder, characterized by degeneration and death of dopaminergic (DA) neurons in the *substantia nigra pars compacta* (SNc) of the ventral midbrain (Moore et al., 2005). The initial link between PD and deregulation of histone acetylation came from observations that the PD linked presynaptic protein, α-Synuclein (α-Syn), binds histones and as a result inactivates HATs like CBP, p300 and PCAF, causing apoptosis in human neuroblastoma cells (Kontopoulos et al., 2006). More recently, the ability of the class I HDACi valproic acid to increase histone acetylation in a rat model of Parkinson's disease was associated with decrease in monoubiquitination of α-Syn that facilitates its nuclear translocation, an increase in tyrosine hydroxylase in both the substantia nigra and striatum as well as prevention of neuronal death in the substantia nigra (Monti et al., 2010). A robust age-related increase in α-synuclein protein within individual nigral neurons has been observed with optical densitometry studies and is strongly associated with age-related decreases in tyrosine hydroxylase (TH), the rate limiting enzyme for dopamine production (Chu and Kordower, 2007). Sirtuins 2 (SIRT2) is one of the seven NAD^+^-dependent class III HDACs (Blander and Guarente, 2004) that functions as α-tubulin deacetylase (North et al., 2003). Mounting evidence indicate that excess SIRT2 might be deleterious to neurons (Suzuki and Koike, 2007; Pfister et al., 2008) and a recent study by Maxwell et al. (2011) also revealed an age-dependent accumulation of SIRT2 in mouse brain and spinal cord and correlate with reduced α-tubulin acetylation in primary mouse cortical neurons. Genetic or pharmacological inhibition of SIRT2 has been reported to rescue α-synuclein toxicity in dopaminergic neurons and in an *in vivo* fly model (Outeiro et al., 2007). The same study also reported that inhibition of SIRT2 in human neuroglioma cells (H4) abates α-synuclein toxicity by promoting formation of enlarged α-synuclein inclusions. Although α-synuclein inclusions are considered a pathological feature of PD, formation of such inclusions likely lowers the concentration of toxic α-synuclein oligomers, reduces aberrant interaction of components of inclusions with cellular proteins and thus has been proposed to have cytoprotective effects (Tanaka et al., 2004). The exact mechanism whereby SIRT2 inhibition affects α-synuclein aggregation remains uncertain. However, α-synuclein has been reported to interact with α-tubulin as well as the microtubule-binding proteins MABP1 and tau (Jensen et al., 2000; Alim et al., 2004). Thus, one possibility is that the increase in acetylated α-tubulin resulting from SIRT2 inhibition may stimulate aggregation of α-synuclein through its affinity to microtubules. Moreover, microtubule stabilization itself could be an important factor contributing to neuroprotection (Outeiro et al., 2007). Together, these studies provide a link between α-synuclein activity, histone deacetylation, neurodegeneration, and aging as well as identify SIRT2 as a potential target for therapeutic intervention in PD.

### Amylotrophic lateral sclerosis (ALS)

The efficacy of restoring histone acetylation levels has also been investigated in Amylotrophic lateral sclerosis (ALS) using HDACi treatments as transcriptional dysregulation is thought to play a role in the disease pathophysiology (Oates and Pamphlett, 2007; Figueroa-Romero et al., 2012). ALS is an adult-onset neurodegenerative disease characterized by progressive loss of motor neurons in the brain, brain stem, and spinal cord, resulting in generalized weakness, muscle atrophy, paralysis, and eventual mortality (Chou, 1997; Ikemoto et al., 2000). ALS has been attributed to gain-of-function mutations in the gene encoding Cu/Zn superoxide dismutase 1 (SOD1) (Orrell et al., 1995). In a SOD1 point mutation mouse model of ALS, ALS symptoms were molecularly accompanied by reduced CBP levels in motorneurons (Rouaux et al., 2003). Treatment of SOD1 mutant mice with HDACi like Sodium Valporate (VPA) significantly suppressed the death of motor neurons, restores the loss of CBP and histone acetylation deficits although it did not prolong survival (Rouaux et al., 2007). Similarly, treating SOD1 mutant mice with 4-phenylbutyrate starting before or shortly after onset of symptoms extends survival and improves pathological phenotypes (Ryu et al., 2005). This study also found that 4-phenylbutyrate treatment ameliorates hypoacetylation, upregulated Bcl-2, NF-κB, p50 and phospho-IκB, and downregulates cytochrome c caspases in the spinal tissues of treated mice. Further evidence for a deregulation of histone acetylation in ALS comes from a recent human postmortem study. Comparing the mRNA expression levels of all class I, II, and IV HDACs in the ALS brain and spinal cord, this study found that both mRNA and protein levels of HDAC2 and HDAC11 were up- and down-regulated, respectively (Janssen et al., 2010). The functional consequences in terms of histone acetylation changes and resulting gene expression changes however, remain unclear.

### Alzheimer's disease

Alzheimer's disease (AD) is the most common form of neurodegenerative disorder and dementia in the elderly. AD manifests itself on the pathological background of amyloid beta (Aβ) plaques, neurofibrillary tangles (NFTs) resulting from intraneuronal aggregates of the microtubule-associated protein, *tau*, and neuronal cell death. Accumulating evidence indicate that signaling between neurons is interrupted at early stages of AD (Trinchese et al., 2008) and recent studies identify dysregulation of epigenetic control mechanisms and the resultant aberrant epigenetic marks as contributing factors to such early neuronal dysfunction (Wang et al., 2008; Mastroeni et al., 2011). A number of different epigenetic abnormalities like DNA methylation [reviewed in Irier and Jin (2012)], phosphorylation (Ogawa et al., 2003), and histone acetylation (Francis et al., 2009) have also been reported in AD (Mastroeni et al., 2011). Further evidence linking histone acetylation and cognitive decline in AD stems from the observation that histone acetylation declines in mouse models for AD. For example, decreased acetylation of H4 but not H3 has been observed in tg2576 mice, a model for amyloid pathology (Ricobaraza et al., 2009). Interestingly, administration of the pan-HDACi phenylbutyrate has been reported to reinstate associative memory and synaptic plasticity in 6- and 16-month old tg2576 mice (Ricobaraza et al., 2011). Similarly, administration of various pan-HDACi also reinstates associative memory in APP/PS1Δ 9 mice, also a mouse model for amyloid pathology (Kilgore et al., 2010). The pan-HDACi TSA has also been reported to restore associative memory function in hippocampal LTP in another mouse model for AD-like amyloid pathology (APP/PS1) that exhibit impaired H4 acetylation upon exposure to a learning stimulus (Francis et al., 2009).

Recent studies have also implicated specific HATs and HDACs in AD associated pathophysiology. Donmez et al. (2010) showed that over-expression of SIRT1, the NAD^+^-dependent deacetylase in a mouse model of AD reduces the production of Aβ and formation of plaques via activation of transcription of the gene encoding α-secretase. Additionally, p25/Cdk5, a kinase complex implicated in AD and other neurodegenerative disorders inhibits HDAC1 in primary rat cortical neurons, rendering these neurons susceptible to DNA damage, cell cycle reentry, and ultimately cell death (Marambaud et al., 2003). Remarkably, overexpression of HDAC1in primary rat cortical neurons rescues such p25/Cdk5-mediated DNA damage and neurotoxicity (Kim et al., 2008). While these findings suggest that AD could be a disease of aberrantly increased histone acetylation, a substantial body of evidence also supports the notion that inhibition of HDACs can be protective and beneficial in AD. In fact, APP overexpression in cultured cortical neurons leads to H3 and H4 hypoacetylation, and is paralleled by decreased CBP levels (Rouaux et al., 2003). Loss of function mutations in genes coding for PS1 and PS2 has been shown to reduce expression of CBP and CBP/CREB target genes such as *c-fos* and BDNF with negative effects on synaptic plasticity, spatial, and contextual memory (Saura et al., 2004). Moreover, in the p25/Cdk5 model of neurodegeneration, treatment with the broad HDACi sodium butyrate not only increased H3 and H4 acetylation levels, but also resulted in the reestablishment of learning abilities, as well as access to long-term memories that had been ablated by prior hyperactivation of p25/Cdk5 (Fischer et al., 2007). Similarly, both general and class I-selective HDAC inhibitors have been shown to ameliorate cognitive defects in transgenic AD mouse harboring hereditary AD mutation (Kilgore et al., 2010; Ricobaraza et al., 2011).

**Figure 2:**
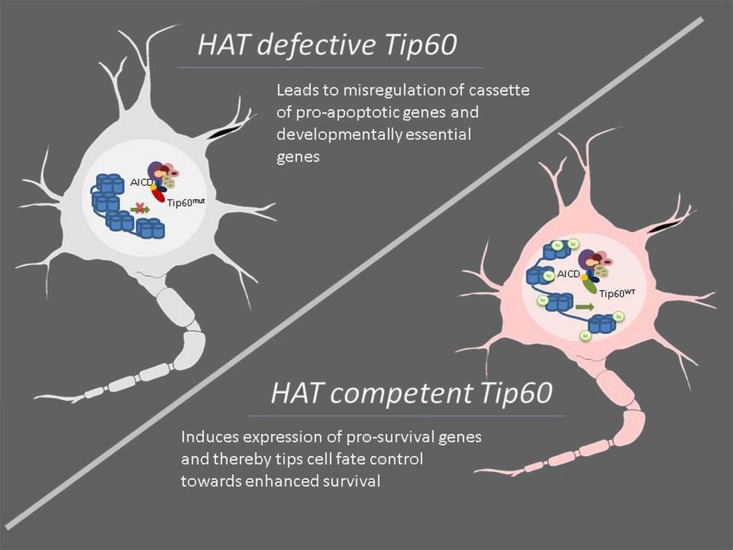
**Modulation of specific HAT function such as Tip60 displays neuroprotective effects under neurodegenerative conditions.** Neurodegenerative diseases are characterized by impaired acetylation homeostasis that consequently leads to altered neuronal transcription profiles, resulting in attenuated expression of survival-associated genes while simultaneously accentuating more pro-apoptotic genes. Conversely, modulation of cellular levels and/or enzymatic activity of specific HATs may enhance the expression of cassettes of specific genes that have neuroprotective effects as evidenced in the case of Tip60. Under amyloid precursor protein (APP) induced neurodegenerative conditions, HAT competent Tip60 (Tip60^WT^) but not its HAT defective counterpart (Tip60^mut^) exerts neuroprotective defects by complexing with the APP intracellular and epigenetically regulating gene expression profiles essential for tipping the cell fate control balance in favor of cell survival. Thus, targeting specific HATs for therapeutic intervention may offer more promising alternatives for neurodegenerative diseases than currently available HDAC inhibitors.

The HAT Tip60 has also been implicated in AD *via* its interaction with the APP intracellular domain (AICD), a fragment that is generated by the sequential processing of APP by β- and γ-secretases and is subsequently released into the cytoplasm (Muller et al., 2008). AICD has been shown to form a transcriptional competent protein complex with the HAT Tip60 via the scaffolding protein Fe65 (Cao and Sudhof, 2001). It has been demonstrated that this complex is recruited to the promoters of certain target genes where it acts to acetylate select histone proteins to epigenetically regulate gene transcription (Cao and Sudhof, 2001; von Rotz et al., 2004; Ryan and Pimplikar, 2005). Importantly, aberrant expression of some of these genes like LRP1, GSK-3B, KAI-1 has been linked to AD pathophysiology (Baek et al., 2002; Muller et al., 2007; Slomnicki and Lesniak, 2008). Based on these findings, it has been proposed that the inappropriate AICD/Tip60 complex formation and/or recruitment may contribute or lead to AD pathology via misregulation of target genes required for neuronal functions. In support of this concept, we recently reported that co-expression of APP with HAT activity deficient Tip60 leads to misregulation of a number of pro-apoptotic genes in a *Drosophila* AD model with a resultant increase in neuronal apoptotic cell death. In contrast, expressing HAT competent wild type Tip60 in conjunction with APP led to enhanced repression of a “cassette” of pro-apoptotic genes along with induction of pro-survival genes like *Drosophila* Bcl-2 that results in a concomitant reduction in neuronal apoptosis. These findings point to the fact that Tip60 may play a neuroprotective role during disease progression via its histone acetylase function (Figure 2). By complexing with the AICD region of APP, Tip60 may epigenetically regulate transcription of genes essential for tipping the cell fate control balance from apoptotic cell death toward cell survival under APP induced neurodegenerative conditions (Pirooznia et al., 2012b). Together, these studies suggest that the overall misregulation of histone acetylation characteristic of AD is complex. While the beneficial effects observed with general or partially selective HDACi are promising, it is essential to identify the specific HATs and HDACs that can be targeted for therapeutic interventions.

## Perspectives on use of HDAC inhibitors for treatment of neurodegenerative diseases

As described above, the promising effects observed with the use of small molecule HDAC inhibitors has ignited enormous interest in their therapeutic potential for various neurodegenerative conditions. However, most HDAC inhibitors that have been tested in the context of neurodegenerative diseases are non-selective, inhibit multiple HDAC proteins, and the observed therapeutic effects likely result from increased “global” and non-specific acetylation levels (Kazantsev and Thompson, 2008). These issues have in turn raised widespread speculation about the target specificity of HDAC inhibitors (Selvi et al., 2010). Recent targeted gene deletion studies indicate that HDACs serve very distinct functions within the adult brain. Cellular localization and tissue-specific expression for different HDACs also vary (Fischer et al., 2010). Broide et al. (2007) recently reported that under native conditions, all HDACs are expressed in the adult rodent brain. However, expression level of HDAC10 is very low under native conditions and can be detected only in the hippocampal formation. In some instances, interactions between different HDAC classes are required to activate their deacetylase function. For example, HDACs 4, 5, and 7 (class II HDACs) lack the ability to deacetylate histones independently and require interaction with HDAC 3 (class I) to be active (Fischle et al., 2002). Contrarily, while class I HDACs 1 and 2 form complexes with each other and are often found in the same protein complexes, they appear to serve distinct functions. Global loss of HDAC1 in mice leads to early lethality, suggesting that HDAC2 cannot compensate for the absence of HDAC1 (Lagger et al., 2002). However, mice lacking either HDAC1 or HDAC2 in the central nervous system display no apparent effect on neuronal development while loss of both HDAC 1 and 2 leads to loss of neuronal differentiation (Montgomery et al., 2009). Thus, in addition to their distinct roles in the adult brain, HDAC 1 and 2 appear to have important redundant functions during neuronal development (Montgomery et al., 2009). Distinct as well as complementary roles for HDAC 1 and 2 have also been observed with regards to synapse development. In immature hippocampal neurons, a targeted knockdown of HDAC 1 and 2 increased synaptic activity and synapse numbers. However, in mature neurons, the knockdown of HDAC2 alone decreased synaptic activity, whereas the loss of HDAC1 had no effect (Akhtar et al., 2009). Thus, inhibition of HDAC1 and 2 during development, and HDAC2 in mature brain, may have potential unexpected neurological side effects. HDAC2 knockout in mice has also been shown to enhance learning and memory and synaptic plasticity (Guan et al., 2009).

Although targeting specific classes of HDACs has been perceived as a suitable therapeutic avenue for some neurodegenerative diseases, it can lead to very different and potentially opposing clinical implications. For example, activation and/or overexpression of HDACs 2 and 3 is associated with neurodegenerative diseases such as ALS and neural cell toxicity (Janssen et al., 2010; Bardai and D'Mello, 2011) whereas both neuroprotective and neurotoxic effects have been described for HDAC1. While inhibition of HDAC1 in CK-p25 mouse model of ischemia has been found to induce DNA damage and cell death (Kim et al., 2008), elevated HDAC1 levels have been observed in brain regions vulnerable to neurodegeneration in two mouse models of neurodegeneration: the striatum of R6/2 model of Huntington's disease and in the cortex and hippocampus of CaMK/p25 double-transgenic mouse model of tauopathic degeneration, thereby suggesting a role for HDAC1 in promoting neuronal death. Elevating HDAC1 expression by ectopic expression also promotes the death of otherwise healthy CGN and cortical neurons in culture, an effect that requires interaction between HDAC1 and HDAC3 (Bardai et al., 2012). The expression of histone deacetylase-related protein (HDRP), a truncated form of HDAC9 generated by alternative splicing and completely lacking the HDAC catalytic domain, is high in healthy neurons but is sharply downregulated in neurons primed to die (Morrison et al., 2006). Forced suppression of HDRP expression induces death in otherwise healthy CGNs exposed to low potassium (LK) conditions, whereas HDRP overexpression inhibits LK induced neuronal death. Such HDRP-mediated neuroprotection depends on deacetylase activity, which is acquired through interaction with HDAC1, suggesting that HDAC1 can contribute to both the survival and death of neurons depending on whether it interacts with HDRP or HDAC3, respectively (Bardai et al., 2012). Similar to HDRP, overexpression of HDAC4 also displays neuroprotective effects in CGN and inhibits LK induced apoptosis via a mechanism distinct from HDRP (Sananbenesi and Fischer, 2009). While HDRP protects neurons by inhibiting apoptosis associated c-jun expression and inhibiting c-jun N-terminal kinase (JNK) activity via direct interaction (Morrison et al., 2006), HDAC4 represses CDK1 activity and suppresses cell cycle progression (Majdzadeh et al., 2008). HDAC4 also has an essential role in neuronal survival in mouse retina and prolongs photoreceptor survival in mice undergoing retinal degeneration (Chen and Cepko, 2009). Besides HDRP, HDAC7 has also been observed to promote neuronal survival in CGNs exposed LK treatment that induces apoptosis by repressing c-jun expression. HDAC7 mediates such a neuroprotective effect by directly associating with the c-jun promoter in a deacetylase independent manner (Ma and D'Mello, 2011). Other class II members like the microtubule associated deacetylase, HDAC6 has also been reported to play a role in lowering the steady state level of aberrant proteins thereby mitigating toxicity. In a study by Pandey et al. (2007), HDAC6 was reported to rescue degeneration caused by impairment of the ubiquitin proteasome system in a *Drosophila* model of Spinobulbar muscular atrophy (SBMA), in an autophagy-dependent manner. HDAC6 has also been reported to function in the autophagic clearance of aggregated Htt proteins via retrograde transport on microtubules (Iwata et al., 2005). Thus, it appears like members of class II HDAC can mediate the neuroprotective effects via different mechanisms. Moreover, subcellular localization of HDACs and thus, their ability to repress gene targets is controlled by synaptic activity in neurons. For instance, localization of class II HDACs 4 and 5 is dynamic and signal-regulated in cultured hippocampal neurons wherein nuclear export of HDAC4 is initiated by spontaneous electrical activity and HDAC5 translocation to nucleus is induced by stimulation of calcium flux through synaptic NMDA receptors (Chawla et al., 2003). Such activity-dependent regulation of HDAC function further necessitates a clearer understanding of specific activating stimuli if pharmacological interventions targeting these HDACs are to be developed. Together with studies demonstrating opposing as well as redundant functions of members of class I HDACs and their requirement for activation of other HDACs, the above studies ascribing neuroprotective roles for specific members of class II HDAC suggest that targeting specific HDACs might be more beneficial than class specific modulation of HDAC activity.

Another issue to consider in terms of HDAC based therapeutic efficacy is that although HDAC inhibitors are generally considered to promote neuronal growth and differentiation, they also exhibit toxicity in various cell types of the central nervous system. For instance, there is evidence that they could have potentially detrimental effects on the orderly maturation of astrocytes and oligodendrocytes (Hsieh et al., 2004; Liu and Casaccia, 2010; Pedre et al., 2011). There is also evidence that neuroprotection can result from non-enzymatic activity of HDACs, as was demonstrated in the case of a mutated inactive form of SIRT1 that prevents apoptosis when overexpressed in cerebellar granule neurons (CGNs) (Pfister et al., 2008). Moreover, like their counterparts, the HATs—class I, II, and III of HDACs also regulate lysine acetylation of non-histone proteins that exert neuroprotective effects (Dokmanovic and Marks, 2005) adding a further layer of complexity to the interpretation of therapeutic potentials of currently available broad spectrum or even class specific HDAC inhibitors for neurodegenerative diseases. Thus, the specificity and side-effect profiles of inhibitors of HDACs require additional investigation to fully gauge their neuroprotective abilities. Further exploration of isoform-selective HDAC inhibitors that are also region-specific may provide a therapeutic advantage in targeting specific cell and tissue functions under pathological conditions.

## Modulating HAT function: a promising therapeutic option for neurodegenerative diseases?

It has become increasingly clear that chromatin acetylation status can be impaired during the lifetime of neurons through loss of function of specific HATs with deleterious consequences on neuronal function (Selvi et al., 2010). Once the acetylation balance is disturbed by the loss of HAT dose, the HAT: HDAC ratio tilts in favor of HDACs in terms of availability and enzymatic functionality, a fact highlighted by amelioration of several neurodegenerative conditions by various HDAC inhibitors (Ittner and Gotz, 2011). In fact, a clue to explain the net deacetylation observed during neurodegeneration came with the finding that dying neurons exhibit progressive loss of HAT activity and/or expression, particularly that of CREB binding protein (CBP) and to a lesser extent p300. Notably, overexpression of CBP under apoptotic conditions delays neuronal cell death, an event that was dependent on the HAT function of CBP (Orrell et al., 1995; Anne-Laurence et al., 2007)

Specific HATs are also emerging as regulators that gate access to genes regulating specific neuronal processes that are essential for maintaining neuronal health and for mediating higher order brain functions. Notably, such processes are also affected in neurodegenerative conditions and significantly contribute to pathological consequences. For instance, CBP has been shown to mediate specific forms of hippocampal long term potentiation, a form of synaptic plasticity thought to underlie memory storage (Wood et al., 2005). In contrast, the HAT p300 has been shown to constrain synaptic plasticity in the prefrontal cortex and reduced function of this HAT is required for formation of fear extinction memory (Marek et al., 2011). Importantly, overexpression of p300 but not HDAC inhibition has been shown to promote axonal regeneration in mature retinal ganglion cells following optic nerve injury, an effect mediated by p300 induced hyperacetylation of histone H3 and p53 that consequently leads to increased expression of selected pro-axonal outgrowth genes (Gaub et al., 2011). Overexpression of Tip60 under APP induced neurodegenerative conditions also induces intrinsic axonal arborization of the *Drosophila* small ventrolateral neurons, a well characterized model system for studying axonal growth (Pirooznia et al., 2012a). It is important to note that modulation of specific HAT levels and/or activity may alter the expression of many genes or “cassettes” of specific genes that act together produce a neuroprotective effect such as that observed in the case of Tip60 (Pirooznia et al., 2012b). While such genes can together produce neuroprotective effects, the same situation might also stimulate expression of death inducing genes as well as detrimental effectors of specific neuronal processes such as that evidenced in studies that overexpress HATs like CBP and Tip60. Therefore, it is essential to determine the identity of specific gene targets regulated by HATs that are enriched for neuronal functions and further dissect the neuroprotective or neurodetrimental effects of such genes in order to devise HAT based therapeutic strategies. With regards to non-chromatin associated cellular processes, the acetyltransferase Elp3 known to acetylate microtubules has been shown to be involved in the regulation of synaptic bouton expansion during neurogenesis (Singh et al., 2010) and recent studies suggest that regulation of microtubule acetylation by the ELP3 might be commonly affected in neurological diseases making it a potential target for acetylation modulator based therapies [reviewed in Nguyen et al. (2010)]. Tip60 has also been recently shown to play a causative role in synaptic plasticity in the *Drosophila* neuromuscular junction partly through acetylation of microtubules (Sarthi and Elefant, 2011). Together, these studies raise the possibility that modulation of expression levels and/or activity of specific HATs such as CBP and Tip60 could be an alternative therapeutic option for neurological conditions.

Importantly, targeting HATs rather than HDACs can also be beneficial because unlike HDACs, HATs have non-redundant functions under physiological conditions and thus the presence of these specific modulators can have more direct effects. In a study by Hoshino et al. (2003), it was reported that the total protein amount and activity of various HDACs is not altered by mutant huntington protein expression in rat primary cortical neurons. Thus, the neurodegeneration associated tilt in HAT: HDAC does not appear to include augmentation of HDAC protein level. Therefore, activation of specific HATs may restore acetylation balance in addition to activating specific gene expression programs that consequently have neuroprotective effects. In fact, a number of recent studies conclude that HDAC inhibitor induced hyperacetylation alone may not be sufficient to produce beneficial effects. In a study by Langley et al. (2005), it was reported that HDAC inhibition mediated enhancement of synaptic plasticity and hippocampus-dependent memory formation requires the presence of at least one wild type allele of *cbp* highlighting the requirement of HATs like CBP for site specific acetylation and the recruitment of the basal transcriptional machinery. However, increasing neuronal dosage of specific HATs to reinstate acetylation homeostasis calls for the same concern as does the utilization of HDAC inhibitors. Non-specific enhancement of HAT levels and/or activity may lead to further complications by skewing the acetylation balance in the neighboring cell population toward hyperacetylation. Therefore, in order to reap the full potential of specific HAT activators, it is also essential to quantify HAT-HDAC dose in specific cell populations that are vulnerable to different degenerative etiologies (Saha and Pahan, 2006).

## Conclusion

In summary, histone acetylation is now recognized as one of the key mechanisms that regulate gene expression programs critical for high-order brain functions like, such as learning and memory. While dynamic yet controlled regulation of histone acetylation and deacetylation is crucial for these functions, deregulation of the system may lead to complex changes in the epigenetic landscape that impairs cognitive functions. Chronic deregulation of the acetylation machinery can ultimately lead to neuronal death and brain atrophy as manifested in neurodegenerative diseases. Clearly, more research is required to fully understand the precise mechanism(s) by which this system impacts neuronal survival and mediates memory functions. This knowledge can then be translated to novel HAT/HDAC based therapeutic strategies for the early intervention of neurodegenerative diseases. However, a major challenge with utilization of modifiers of cellular acetylation levels is the identification of *bona fide* targets of HATs and HDACs and the integration of histone and transcription factor acetylation into a broader context of neuronal, and importantly, cellular homeostasis (Langley et al., 2005). Although still in its infancy, the neuroprotective effects displayed by HATs like CBP, p300 and Tip60 and specificity of these effects for particular neuronal processes appears more promising than currently available non-selective HDAC inhibitors. However, determining the genes or “cassettes” of genes that are regulated by such HATs and characterizing the survival or degenerative effects such genes have would subsequently facilitate the development of novel drugs and specific therapeutic strategies with lower adverse side effects than those currently available.

### Conflict of interest statement

The authors declare that the research was conducted in the absence of any commercial or financial relationships that could be construed as a potential conflict of interest.
